# Quantitative susceptibility mapping at 7T as a biomarker of post- and interictal extravascular iron in patients with focal epilepsy

**DOI:** 10.1016/j.ebiom.2025.106040

**Published:** 2025-11-27

**Authors:** Nina Rebecca Held, Tobias Bauer, Rüdiger Stirnberg, Theda von der Recke, Nils Christian Lehnen, Tobias Baumgartner, Juri-Alexander Witt, Attila Rácz, Jan Pukropski, Lennart Walger, Randi von Wrede, Mostafa Badr, David Wolf, Lennart Nils Kersting, Annalena Lange, Justus Bisten, Markus Schmidt, Eberhard Pracht, Daniel Löwen, Jennifer Faber, Christoph Helmstaedter, Martin Reuter, Alon Friedman, Alexander Radbruch, Rainer Surges, Tony Stöcker, Theodor Rüber

**Affiliations:** a Department of Neuroradiology, University Hospital Bonn, Bonn, Germany; b Department of Epileptology, University Hospital Bonn, Bonn, Germany; c German Centre for Neurodegenerative Diseases (DZNE), Bonn, Germany; d Institute for Computer Science, University of Bonn, Bonn, Germany; e Center for Neurology, Department of Parkinson's Disease, Sleep and Movement Disorders, University Hospital Bonn, Bonn, Germany; f A.A. Martinos Centre for Biomedical Imaging, Massachusetts General Hospital, Boston, MA, United States; g Department of Radiology, Harvard Medical School, Boston, MA, United States; h Department of Brain and Cognitive Sciences, The School of Brain Sciences and Cognition, Zlotowski Centre for Neuroscience, Ben-Gurion University of the Negev, Beer-Sheva, Israel; i Department of Physiology and Cell Biology, Faculty of Health Sciences, Ben-Gurion University of the Negev, Beer-Sheva, Israel; j Department of Medical Neuroscience, Dalhousie University, Halifax, Canada; k Center for Medical Data Usability and Translation, University of Bonn, Bonn, Germany; l Department of Physics and Astronomy, University of Bonn, Bonn, Germany

**Keywords:** Ultra-high-field MRI, Blood–brain-barrier dysfunction, Postictal imaging, Neuroinflammation

## Abstract

**Background:**

Iron deposition in the brain has been associated with epilepsy, but its anatomical distribution and its temporal association to seizures remain unclear. Here, we investigate brain iron in individuals with focal epilepsy using interictal and postictal quantitative susceptibility mapping (QSM) at 7 T MRI and explore its functional relevance.

**Methods:**

80 consecutive participants with focal epilepsy undergoing presurgical evaluation and 50 healthy controls were included. 8/80 participants with focal epilepsy had interictal and postictal scans. High-resolution T1- and susceptibility-weighted images were acquired and processed using custom pipelines for QSM. Seizure onset zones were determined by expert consensus, and neuropsychological assessments included memory and mood. Parcel-wise analyses examined group differences and correlations with cognitive scores, controlling for age and sex.

**Findings:**

Participants with focal epilepsy showed significantly higher interictal susceptibility, which colocalised with the presumed seizure onset zone. Impaired memory performance and more severe depressive symptoms were correlated with higher interictal susceptibility. Postictal susceptibility was significantly higher than interictal susceptibility and associated to the number of preceding seizures.

**Interpretation:**

QSM reflects both ictal iron extravasation and interictal iron accumulation. If further validated, it may help to non-invasively delineate the epileptogenic zone and become a long-term biomarker for seizure-burden.

**Funding:**

N.R.H. and T.Bauer were supported by the BONFOR research commission of the Medical Faculty of the University of Bonn. T.Bauer was funded by the 10.13039/501100001659Deutsche Forschungsgemeinschaft. T.Bauer., L.W., A.L. and T.R. are funded by the German Ministry for Research, Technology and Space.

**Research in context**

**Evidence before this study**

Emerging evidence suggests that dysregulated brain iron homoeostasis plays a significant role in the pathophysiology of epilepsy. Accumulation of iron has been observed in resected brain tissue from patients with epilepsy and *in vivo* using susceptibility-weighted imaging, particularly in regions near seizure onset zones. Experimental models indicate that iron contributes to epileptogenesis via multiple mechanisms, including oxidative stress, neuroinflammation, ferroptosis and enhanced neuronal excitability. Dysfunction of the blood–brain barrier is a key contributor to this process, allowing iron-rich blood components to enter the brain parenchyma, particularly after seizures. However, other mechanisms, such as seizure-induced microvascular injury, mitochondrial dysfunction, impaired iron storage and clearance, and altered glial activity, may also drive iron dysregulation. Quantitative susceptibility mapping is an advanced MRI technique that can detect changes in magnetic susceptibility related to iron. However, its functional relevance and temporal dynamics in epilepsy remain poorly understood.

**Added value of this study**

This study is the first to use ultra-high-field (7 T) Quantitative Susceptibility Mapping to investigate interictal and postictal iron-related susceptibility changes in a large, well-characterised cohort of participants with focal epilepsy. We demonstrate that susceptibility is elevated throughout the brain in epilepsy and aligns spatially with seizure onset zones. These elevations are not static; postictal scans reveal additional increases in susceptibility proportional to recent seizure activity. Importantly, increased susceptibility correlates with impaired memory and more severe depressive symptoms, underscoring its clinical relevance. By linking these changes to acute (seizure-related) and chronic (interictal) processes and identifying their associations with cognitive and affective function, our study suggests that iron accumulation may serve as a functionally relevant indicator of disease burden in epilepsy.

**Implications of all the available evidence**

This study uses quantitative susceptibility mapping to investigate seizure-related changes in brain iron, thereby refining our understanding of the anatomical distribution and clinical relevance of iron accumulation in epilepsy. Our findings suggest that Quantitative Susceptibility Mapping may serve as a non-invasive tool for assessing the anatomical localisation of epileptic activity and its broader systemic consequences. Beyond localisation, susceptibility-based imaging biomarkers could help quantify seizure burden and inform prognosis, particularly in relation to cognitive and psychiatric comorbidities. Consequently, with further validation Quantitative Susceptibility Mapping could potentially complement existing clinical and imaging modalities for epilepsy, providing valuable insights into epileptogenic mechanisms and downstream functional impairment.

## Introduction

Epilepsy affects over 50 million individuals worldwide and is characterised by recurrent, unprovoked seizures arising from hyperexcitable neural networks.^1^ While its aetiologies are diverse, converging evidence points to a role for dysregulated brain iron homoeostasis in the pathophysiology of epilepsy.^2^^,^^3^ Iron is essential for normal brain function, yet elevated iron levels within the brain have been associated with several pathological processes: They increase neuronal excitability, trigger inflammation through the generation of reactive oxygen species,^4^ and directly induce neurotoxicity, leading to cell death through a process known as ferroptosis,^3^ all lowering the seizure threshold.^5^^,^^6^ In epilepsy, iron accumulation within the brain is primarily facilitated by dysfunction of the blood–brain barrier (BBB). The BBB is a highly selective, semipermeable interface that regulates the exchange between the extracellular matrix of the central nervous system and the blood.^7^ When the BBB becomes compromised, it permits the uncontrolled passage of blood-borne ions and larger molecules, such as iron-rich haemoglobin, into the brain's extracellular matrix.^8^ Additionally, seizure-induced microvascular injury can lead to microbleeds,^9^ releasing haemoglobin-derived iron directly into the tissue. Chronic neuroinflammation further exacerbates iron dysregulation by altering the expression of iron transport and storage proteins, including divalent metal transporter 1, ferritin, and ferroportin.^10^ mitochondrial dysfunction, a consequence of sustained metabolic stress during seizures, may also contribute to intracellular iron release.^11^ Together, these mechanisms create a pro-oxidative environment conducive to epileptogenesis. Besides, it has been shown that individual seizures are temporally and anatomically related to postictal BBB dysfunctions^12^ and that repetitive seizures or status epilepticus precede chronic/interictal BBB dysfunction.^13^, ^14^, ^15^, ^16^ Hence, there may be a positive feedback loop with seizures contributing to epileptogenesis by recurring and progressive BBB dysfunction and iron accumulation.^17^ The clinical and functional relevance of brain iron, however, remains unclear.

Quantitative susceptibility mapping (QSM) is an advanced magnetic resonance imaging (MRI) technique that allows for the *in-vivo* quantification of iron in the brain, specifically in grey matter.^18^ QSM leverages the interactions between paramagnetic and diamagnetic materials and the MRI's magnetic field. A high signal-to-noise ratio and a strong susceptibility contrast is particularly well-achieved at ultra-high field strengths such as 7 T (7T), enabling high-resolution-QSM. Iron deposition, as detected *in-vivo* through QSM, has been identified across a range of neuropsychiatric disorders, including schizophrenia,^19^ depression,^20^ and attention-deficit/hyperactivity disorder.^21^ Notably, increased iron levels have also been observed in the substantia nigra of individuals with Parkinson's disease^22^, ^23^, ^24^ and in the motor cortex of those with amyotrophic lateral sclerosis,^25^ highlighting their presence in regions closely associated with the underlying pathology. In epilepsy, QSM detected iron deposition in the hippocampus of people with temporal lobe epilepsy^13^^,^^26^ and in the vicinity of the presumed seizure onset zone.^2^^,^^15^ Here, we leverage the potential of ultra-high-field 7T QSM, to investigate interictal iron accumulation and postictal iron extravasation in participants with focal epilepsy. We hypothesised that susceptibility as marker of blood-borne iron in the ultra-high-field is higher in participants with focal epilepsy as compared to healthy controls, anatomically related to the presumed seizure onset zone, functionally relevant, and shows transient peaks related to preceding seizures.

## Methods

### Participants

We prospectively enrolled all individuals with focal epilepsy who underwent presurgical evaluation at the Department of Epileptology, University Hospital Bonn, between June 2023 and June 2024. Inclusion criteria were a clinically confirmed diagnosis of focal epilepsy, age ≥18 years and the ability to undergo an ultra-high-field MRI scan. In all enrolled participants with epilepsy, MRI scans were performed after a seizure-free interval of at least 48 h. If it was clinically reasonable and logistically feasible, an additional postictal MRI examination was performed at the shortest possible interval after the end of a seizure recorded in our telemetry unit. Lastly, we recruited healthy control participants who had no history of neurologic or psychiatric disorders and were ≥18 years old by word-of-mouth advertising and advertisements at our institution. Exclusion criteria for all participants were electrically or mechanically active implants, electrically conductive implants within 6 months after implantation, and ingrown implants after case-by-case decision from medical physics experts.

### Ethics

The study was approved by the institutional review board of the University Hospital Bonn (083/20) and all participants provided written informed consent.

### Delineation of seizure onset zone

For participants with focal epilepsy, five expert raters determined the presumed seizure onset zones based on a multimodal consensus, incorporating seizure semiology, neuropsychological testing, ictal and interictal scalp electroencephalography, and MRI findings; in selected cases, additional modalities such as morphometric analysis program (*n* = 8), ^18^F-fluorodeoxyglucose positron emission tomography (*n* = 65), stereo-electroencephalography (*n* = 17), or ictal single-photon emission computed tomography (*n* = 14) were also considered. Multiple choices were permitted. A presumed seizure onset zone was included if it received a majority vote from at least three out of the five raters. Inter-rater reliability was assessed using pairwise agreement of at least one matching hemisphere-lobe combination between raters and Krippendorff's alpha.^27^

### T1-weighted image acquisition

Whole-brain structural T1-weighted images were acquired at 0·6 mm isotropic resolution using a custom MPRAGE sequence. Universal GRAPE pulses for excitation and inversion, pre-computed from the same B_1_^+^/B_0_ database were used for a homogeneous contrast throughout the brain. 1 x 2_z1_ CAIPIRINHA (Controlled Aliasing In Parallel Imaging Results IN Higher Acceleration)^28^ parallel imaging was combined with elliptical sampling.^29^ Further parameters: field-of-view (matrix size) = 256·0 x 217·6 x 172·8 mm^3^ (428 x 364 x 288), sagittal slice orientation, readout bandwidth = 433 Hz/mm, TE/TI = 2·71 ms/1100 ms, nominal flip angle = 5°, TA = 7:24 min. Furthermore, the scan protocol comprised a fast, 3D-gradient-echo-based, whole-head B_0_ field mapping sequence (3 mm isotropic, elliptical sampling, field-of-view = 243 x 228 × 180 mm^3^, sagittal slice orientation, readout bandwidth = 713 Hz/mm, TE_1_/TE_2_/TR = 0·75 ms/1·94 ms/5 ms, TA = 19 s). All CAIPIRINHA-accelerated images were reconstructed online using the vendor 3D GRAPPA (Generalized Auto-calibrating Partially Parallel Acquisition)^30^ image reconstruction program (“IcePAT”) with complex-valued adaptive coil combination using a virtual reference coil method.

### T1-weighted pre-processing

Pre-processing of T1-weighted images included denoising with ANTs and an intense bias field correction, that was performed with SPM12^31^ with a full width at half maximum (FWHM) of 18 mm, a sampling distance of 2, and only very light regularisation of 10^−4^. While these settings result in slow bias correction, they are efficient in dealing with the residual bias fields encountered at 7T despite the use parallel transmission to mitigate B_1_^+^ inhomogeneity. Pre-processed T1-weighted images underwent atlas-based segmentation and cortical parcellation using FreeSurfer 7·4·1.^32^^,^^33^

### Susceptibility-weighted image acquisition

High-resolution susceptibility-weighted 3D gradient echo images were acquired at 7T (MAGNETOM 7TPlus, Siemens Healthineers, Erlangen, Germany) using a custom skipped-CAIPI (segmented k-space blipped-Controlled Aliasing In Parallel Imaging) 3D-EPI (3D echo planar imaging) sequence.^34^ CAIPIRINHA 1 x 3_z1_ parallel imaging and an EPI factor of 17 result in a total acquisition time of 5:38 min for two whole-brain dual-polarity averages at 0·4 mm isotropic resolution.^35^ Transmit field (B1) inhomogeneities may degrade image quality at 7T in eccentric infratentorial brain regions, which limits applications in temporal lobe epilepsy,^36^^,^^37^ however, in this study we successfully mitigated B1 inhomogeneities by using custom parallel transmission universal pulses.^38^ Parallel transmission was used for a homogeneous excitation flip angle of 13° (nominal) by means of a universal pulse. The particular universal binomial-11 GRAPE^39^ (Gradient Ascent Pulse Engineering) water excitation pulse (1.03 ms duration) was pre-computed on a B_1_^+^ and B_0_ map database of 30 healthy volunteers using an 8-channel transmit/32-channel receive head coil (Nova Medical, Wilmington, USA). Further protocol parameters were: field-of-view (matrix size) = 204·0 x 204·0 x 165·6 mm^3^ (510 x 510 x 414), sagittal slice orientation (anteroposterior phase encoding), readout/phase encode bandwidth = 1630 Hz/mm/81 Hz/mm, TE/TR = 19 ms/40 ms (Echo time/Repetition Time).

### Susceptibility-weighted imaging pre-processing

The two dual-polarity averages of susceptibility-weighted data were (1) co-registered using FSL (mcflirt, applyxfm4d), (2) phase-matched, (3) averaged and (4) denoised using ANTs (non-local means denoising) as explained in a previous publication.^35^ All steps were performed retrospectively on the complex-valued image data (real/imaginary or magnitude/phase representation, where applicable) using a custom nipype pipeline.^40^

### Quantitative susceptibility mapping

**Fig. 1:**
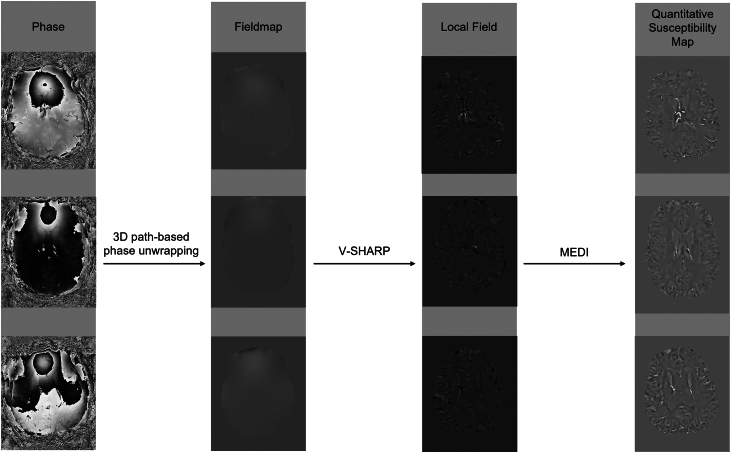
**QSM processing workflow.** Raw phase images were unwrapped using three-dimensional path-based phase unwrapping, then local fields were extracted using the variable-kernel sophisticated harmonic artefact reduction (V-SHARP) algorithm. Final quantitative susceptibility maps were computed with Morphology Enabled Dipole Inversion (MEDI) and are displayed on a scale from −0·2 to 0·2 ppm.

**Fig. 2:**
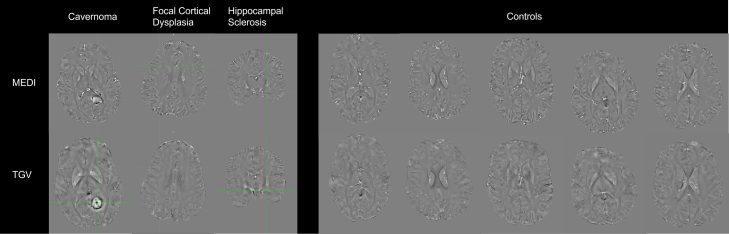
**QSM processing comparisons.** Top: Quantitative Susceptibility Maps using Morphology Enabled Dipole Inversion (MEDI). Bottom: Quantitative Susceptibility Maps using Total Generalized Variation (TGV). QSM are displayed on a scale from −0·2 to 0·2 ppm. From left to right: cavernoma, focal cortical dysplasia, hippocampal sclerosis, five healthy controls.

A custom pipeline was constructed using a MATLAB-based toolbox for quantitative susceptibility mapping in accordance with the recommendations of the ISMRM (International Society for Magnetic Resonance in Medicine) electromagnetic tissue properties study group's 2024 recommendation.^41^^,^^42^ Initially, a brain mask was generated using FSL's Brain extraction Tool (BET) with a fractional intensity threshold of 0·4, determined optimal for our dataset through empirical testing. A three-dimensional path-based phase unwrapping algorithm was employed for total field recovery, known for its robustness in highly wrapped regions.^43^ To compute a weighting map for dipole field inversion, the standard deviation of the field map was inverted and normalised using a threshold defined as the median plus three times the interquartile range. Extreme values above the threshold were replaced with a smoothed version of the weights using a 3 × 3 × 3 voxel filter. This process adjusted the contribution of each voxel to the susceptibility map according to its noise level, reducing artefacts in high-noise regions. Background field contributions were removed using a variable-kernel sophisticated harmonic artefact reduction algorithm.^44^^,^^45^ Residual B_1_-field effects were mitigated through fourth-order polynomial fitting. Morphology Enabled Dipole Inversion (MEDI)^46^ was employed for field-to-source inversion, using a low regularisation parameter (λ = 200) and a high fraction of edge voxels (95%) to reduce oversmoothing while maintaining artifact suppression. MEDI stabilises the inherently ill-posed dipole inversion by encouraging susceptibility map edges to align with the magnitude image, preserving fine anatomical structures. Edge voxels, corresponding to sharp tissue transitions such as grey-white matter boundaries or vessels, are allowed to vary more freely, while non-edge voxels are heavily regularised. Local-field data were k-space zero-padded with an array size of [20 20 20] prior to dipole inversion, in line with established signal processing techniques.^47^, ^48^, ^49^ This approach improves the nonlinear operations required for susceptibility mapping and enhances apparent spatial resolution, enabling more accurate dipole-kernel deconvolution.^50^ Finally, model error was reduced through iterative tuning.^51^ All QSM datasets underwent systematic quality control, including visual inspection for complete brain coverage, absence of motion or ghosting artifacts, and successful registration to structural images. Noise maps were also reviewed to confirm sufficient image quality, and no datasets required exclusion. Step-by-step reconstruction examples are shown in Fig. 1. For additional validation, a subset of datasets was also reconstructed using the Total Generalized Variation (TGV)^52^ method and compared with MEDI (Fig. 2); Bland–Altman analyses across subcortical regions were performed to assess measurement consistency. Magnitude images of the susceptibility-weighted scans were then registered to the FreeSurfer-processed T1-weighted images using a boundary-based cost function and the transformation was applied to the processed quantitative susceptibility map.^53^ We used the left lateral ventricle mask of the FreeSurfer segmentation for zero-referencing of the susceptibility values to allow robust comparison of absolute values between participants.

### Neuropsychological testing

In participants with focal epilepsy, verbal memory was assessed using the verbal learning and memory test (VLMT).^54^ We assessed figural memory employing the revised Diagnosticum fuer Cerebralschaedigung (DCS-R).^55^ Memory scores were standardised according to a normative sample of 488 healthy individuals (mean = 100, standard deviation = 10) and corrected for age. To assess hippocampal function irrespective of the lesional hemisphere, the average of the two test parameters most sensitive for left (delayed free recall trial of the VLMT) and right hippocampal function (DCS-R learning performance across all trials), respectively, was used.^56^^,^^57^ We refer to this parameter as “episodic memory”. For the assessment of mood, Beck's depression inventory (BDI) was used.^58^

### Statistics

Participants with right-hemispheric presumed seizure onset zone were flipped along the left–right axis for parcel-wise analyses, unless lateralised memory function (verbal or figural) was tested. All linear regressions were computed using the *statsmodels* Python package (v0·14·4). The Benjamini-Hochberg procedure was performed to correct for false-discovery rate (FDR).^59^ For parcel-wise comparisons between groups, susceptibility values were used as dependent variable, independent variables were age at MRI, sex and the group distinction (epilepsy vs. control). Post-hoc power analyses were based on parcel-wise *R*^2^ values and *F*-tests. For parcel-wise correlation with neuropsychological test scores, susceptibility values were again used as dependent variable, independent variables were age at MRI, sex and the respective standardised test score. Effect sizes of group differences are expressed as Glass’ delta, individual susceptibility maps relative to the control group are expressed as *z*-scores. We consider *P <* 0·01 significant.

### Role of funders

The funders had no influence on the study design, data collection, data analyses, interpretation, manuscript preparation, or the decision to submit for publication.

## Results

### Study cohort

**Fig. 3:**
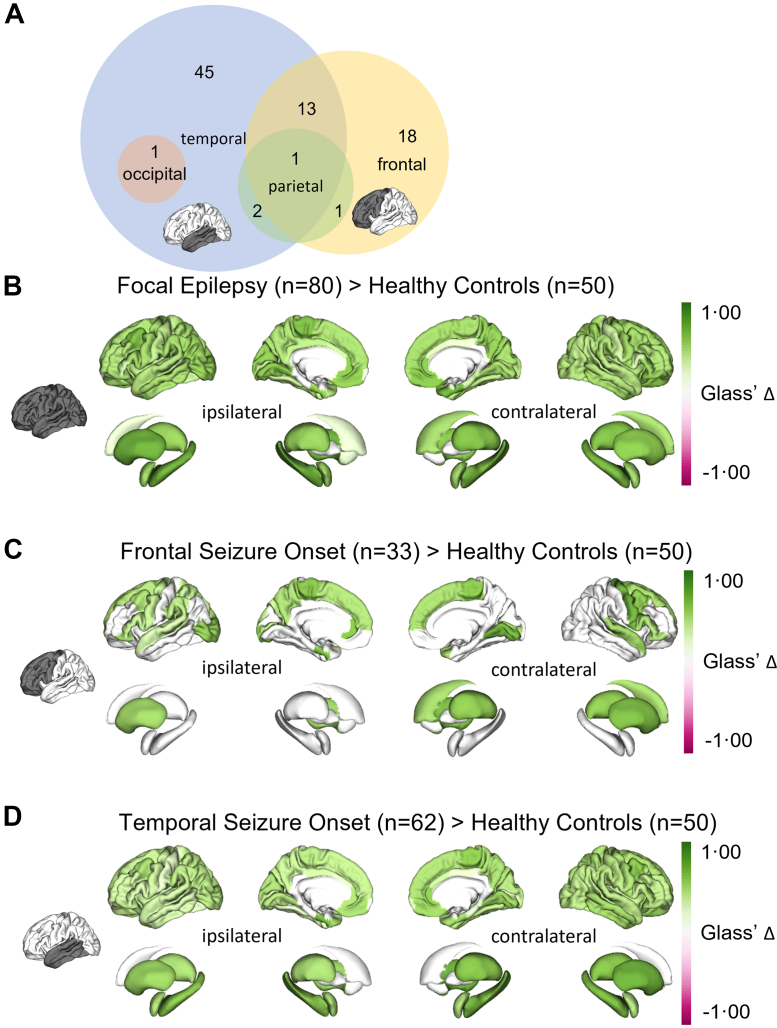
**Group-wise comparisons of interictal susceptibility values.** Group-wise comparisons between individuals with focal epilepsy and healthy controls. Glass' delta of significant (*P* < 0·01, *t*-test) parcels are shown. (A) Venn-diagram of subgroups based on presumed seizure onset zone. (B) Group-wise comparison between all individuals with focal epilepsy (*n* = 80) and healthy controls (*n* = 50). (C) Group-wise comparison between individuals with a frontal presumed seizure onset zone (*n* = 33) and healthy controls (*n* = 50). (D) Group-wise comparison between individuals with a temporal presumed seizure onset zone (*n* = 62) and healthy controls (*n* = 50).

We included 80 participants with focal epilepsy (median age 33 years, range 18–72, 45 male, 35 female, 51 left-hemispheric focus, 29 right-hemispheric focus). A frontal presumed seizure onset zone was determined in 33/80 (41%), a temporal seizure onset zone was observed in 62/80 (78%), 4/80 (5%) had a parietal seizure onset zone and 1/80 (1%) had an occipital seizure onset (Fig. 3A). Inter-rater reliability was assessed using two complementary metrics: a lenient partial-overlap criterion yielding 0·921 pairwise agreement and Krippendorff's alpha of 0·659, indicating moderate agreement.^27^ The cohort included both lesional and non-lesional cases, where ‘non-lesional’ refers to negativity on 3 T MRI performed as part of the diagnostic workup. Structural lesions present in the cohort included focal cortical dysplasias, hippocampal sclerosis, low-grade epilepsy-associated tumours, vascular malformations, and acquired gliotic lesions. Group-wise demographic features are presented in Table 1. The control group consisted of 50 individuals (median age 28 years, range 19–73, 26 male, 24 female). Supplementary Table S1 provides detailed information of all participants with focal epilepsy and healthy controls, including clinical variables, neuropsychological scores, and rating criteria.

**Table 1:** Demographics.

Group	Healthy controls (*n* = 60)	All focal epilepsy (*n* = 80)	Frontal (*n* = 33)	Temporal (*n* = 62)	Parietal (*n* = 4)	Occipital (*n* = 1)
Sex female; *n* (%)	24 (48%)	35 (43%)	9 (27%)	30 (48%)	2 (50%)	1 (100%)
Age; median (range)	28 (19–73)	31 (18–72)	29 (18–55)	30·5 (19–72)	30 (23–55)	30–34
Age at onset; median (range)	n/a	14 (0–67)	12 (1–28)	14·5 (0–67)	12·5 (2–28)	25–30
Verbal memory; *n,* median (range)	n/a	65/80, 93·22 (55·25–110·04)	25/33, 97·95 (66·54–109·36)	52/62, 91·52 (55·25–110·04)	3/4, 97·95 (79·62–98·65)	0/1
Figural memory; *n* median (range)	n/a	67/80, 88·94 (58·65–109·62)	25/33, 84·6 (60·98–107·33)	54/62, 89·455 (58·65–109·62)	4/4, 72·235 (61·17–103·53)	0/1
Episodic memory; *n* median (range)	n/a	60/80, 87·155 (56·2–112·28)	19/33, 93·8 (77·59–109)	49/62, 84·97 (56·2–112·28)	4/4, 82·6925 (69·07–98·48)	0/1
BDI; *n* median (range)	n/a	60/80, 9 (0–45)	19/32, 8 (4–18)	49/62, 9 (0–45)	1/4, 41 (41–41)	0/1
Anti-seizure medication; median (range)	n/a	5 (1–18)	5 (2–12)	5 (1–18)	5·5 (3–12)	3
MRI-negative; *n* (%)	n/a	30 (41%)	20 (60%)	21 (33%)	1 (25%)	0 (0%)
Malformations of cortical development; *n* (%)	n/a	19 (23%)	8 (24%)	12 (19%)	1 (25%)	0 (0%)
Hippocampal Sclerosis; *n* (%)	n/a	10 (12%)	2 (6%)	10 (16%)	1 (25%)	0 (0%)
Low-grade epilepsy-associated tumours; *n* (%)	n/a	8 (10%)	2 (6%)	7 (11%)	1 (25%)	0 (0%)
Vascular malformations; *n* (%)	n/a	9 (11%)	4 (12%)	7 (11%)	0 (0%)	1 (100%)
Acquired gliotic lesions; *n* (%)	n/a	15 (18%)	4 (12%)	13 (20%)	0 (0%)	0 (0%)

BDI: Beck's depression inventory (high scores in the BDI indicate more severe depressive symptoms). In a subset of individuals, more than one structural lesion was present.

### Group-wise comparisons

Across all participants with focal epilepsy (mean power 0·79), significantly (FDR-corrected *P <* 0·01, *t*-test) higher susceptibility values relative to controls were observed in all cortical parcels and all subcortical structures (Fig. 3B). In the subgroup of people with epilepsy and a frontal presumed seizure onset zone (mean power 0·45), we found significantly higher susceptibility values in cortex parcels of all ipsilateral lobes, predominantly the contralateral frontal lobe and both ipsi- and contralateral subcortical structures except the hippocampi and amygdalae (Fig. 3C). People with a temporal presumed seizure onset zone (mean power 0·81) showed significantly higher susceptibility values in all cortical parcels, with larger effects in the ipsilateral hemisphere. In subcortical regions, largest effects of higher susceptibility values were observed for both hippocampi (Fig. 3D). In all above comparisons, no parcels showed significantly lower susceptibility values in participants with focal epilepsy than in healthy controls.

### Neuropsychological associations

**Fig. 4:**
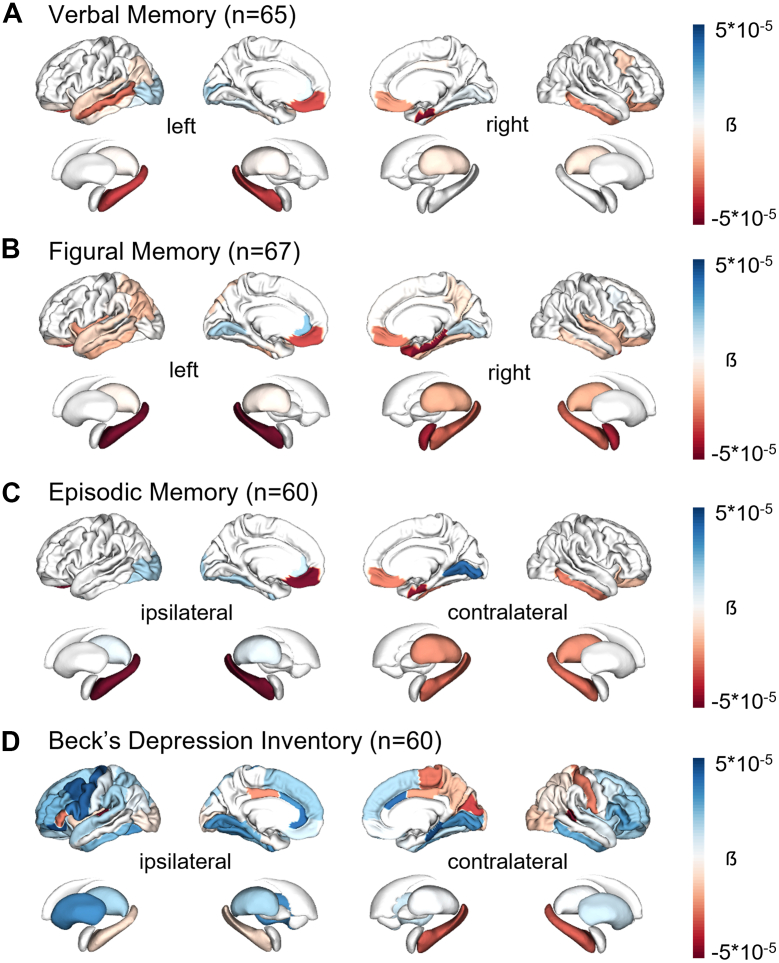
**Associations between interictal susceptibility values and neuropsychological tests.** Regression coefficients of significant (*P* < 0·01, *t*-test) models shown per parcel. (A) Correlation with verbal memory (*n* = 63). (B) Correlation with figural memory (*n* = 65). (C) Correlation with episodic memory (*n* = 60). (D) Correlation with Beck's depression inventory (*n* = 60).

Across all participants with focal epilepsy, poorer verbal memory performance was significantly (FDR-corrected *P <* 0·01, *t*-test) associated with higher susceptibility values of left-temporal cortical parcels and the hippocampus (Fig. 4A). Poorer figural memory performance was significantly correlated with higher susceptibility values of both hippocampi, the right amygdala, right thalamus and bilateral temporal cortical parcels (Fig. 4B). Low scores in the combined hippocampal tests showed strongest associations with high susceptibility values in both hippocampi (Fig. 4C). High scores in the Beck's depression inventory (BDI), indicating more severe depressive symptoms, were significantly correlated with higher susceptibility values in bilateral frontal cortical regions and lower susceptibility values in the contralateral hippocampus and contralateral centroparietal cortical regions (Fig. 4D). No significant differences (all *P* ≥ 0·077, *t*-test) in neuropsychological performance or mood were observed between focal epilepsy subgroups (temporal vs. frontal).

### Postictal cases

**Fig. 5:**
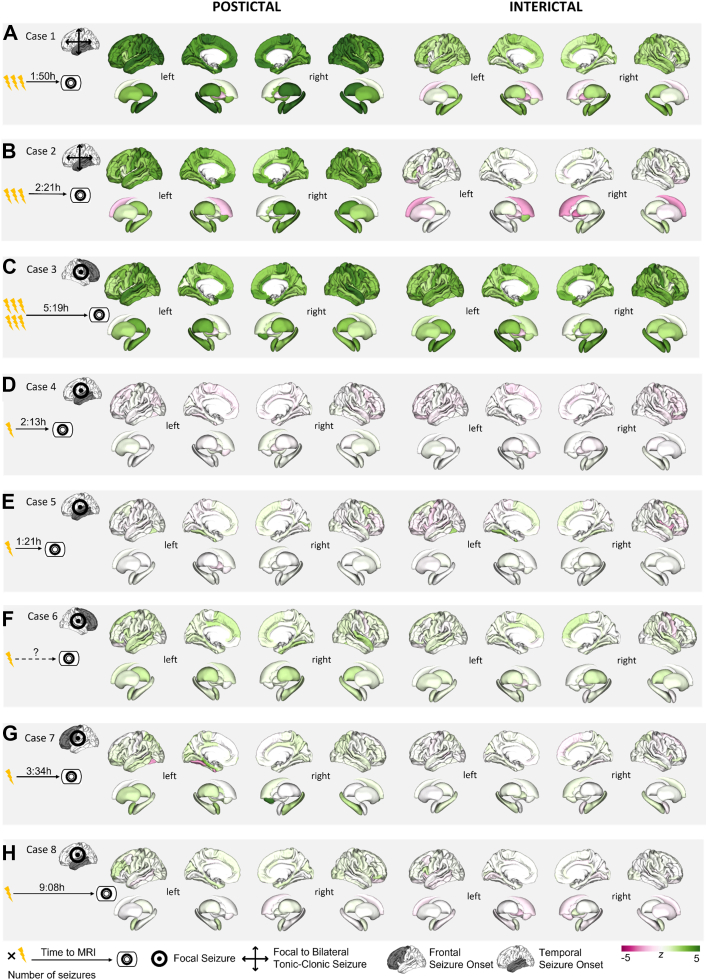
**Individual interictal and postictal susceptibility maps.** All susceptibility values are z-scored relative to healthy controls. Eight cases (A–H) are shown.

In eight cases, we were able to acquire a postictal MRI in addition to the interictal scan (Fig. 5). Details on demographics, aetiology, seizure classification and semiology can be found in Table 2. Most participants after focal to bilateral tonic-clonic seizures (cases 2 and 3, but not case 8) had higher postictal susceptibility values across the entire cortex than participants after focal seizures. Most participants individuals who had a cluster of seizures before the seizure instead of a single seizure had higher susceptibility values also interictally (case 1 and 3, but not case 2). In several cases, the distribution of higher postictal susceptibility values colocalises with potentially epileptogenic MRI abnormalities, for instance in case 8 (hyperintensity of the left amygdala) and in case 2 (volume increase of left amygdala and hippocampus) or with the presumed seizure onset zone in MRI-negative participants as inferred from EEG, clinical and neuropsychological examinations in case 1 and 3 (left temporal).

**Table 2:** Overview over postictal cases.

ID	Age bin (years)	Sex	Time Between seizure onset and mri (h:min)	Aetiology	Seizure classification	Semiology
1	35–39	F	1:50	Unknown	Epilepsy with focal preserved consciousness seizures, focal impaired consciousness seizures, and focal-to-bilateral tonic-clonic seizures	Patient initially awake, lying in bed with eyes closed, sudden seizure onset characterised by: unformed vocalisations (ictal screaming), symmetric hyperkinetic movements, body rocking, facial flush (vegetative sign), patient leaves bed and exits the room, later observed sitting beside the bed: face not visible, altered awareness, abortive generalised clonic movements, patient stands up, then is assisted back into bed, postictal phase: unresponsive, eyes open, subsequently falls asleep, ECG: Ictal tachycardia with heart rate increasing from ∼90 bpm to approximately 150 bpm.
2	30–34	F	2:21	GAD65-antibody associated limbic encephalitis	Epilepsy with focal impaired consciousness seizures (with observable onset, automatisms)	Awake at onset, left hand on the abdomen, fixed gaze, dystonic posturing is observed in the right hand, accompanied by oral automatisms, followed by head and gaze deviation to the left—initially non-forced, becoming forced, culminating in a bilateral tonic-clonic seizure
3	30–34	M	5:19	Unknown	Epilepsy with focal impaired consciousness seizures (impaired awareness, motor onset, hyperkinetic)	Waking up from sleep with a fixed stare, hyperkinetic movements of the left limbs (including throwing things around), unresponsiveness for 13 s, then responding to speech.
4	18–19	F	2:13	Structural (left temporal cavernoma)	Epilepsy with focal seizures (with observable onset, automatisms)	Onset of clinical symptoms 8 s after onset of seizure pattern in EEG. The patient begins to smile and moan. Oral automatisms occur. She speaks incoherently. Disturbances of consciousness.
5	18–19	M	1:21	Unknown	Epilepsy with focal seizures (with observable onset)	Behavioural arrest, nose wiping with the left hand, impaired reaction to speech.
6	30–34	M	Unknown	Structural (right frontal focal cortical dysplasia)	Epilepsy with focal seizures (with observable onset)	Awake, hyperkinetic movements, pedalling for 35 s, immediately reorientated after the end of symptoms.
7	25–29	M	3:34	Structural (left frontal focal cortical dysplasia)	Epilepsy with focal preserved consciousness seizures (with observable onset, hyperkinetic)	Waking up from sleep by opening the eyes, stretching the legs, followed by hyperkinetic movements with rotation around the body axis by 90° to the left, then undirected movements of the limbs.
8	25–29	F	9:08	Structural (left hippocampal sclerosis)	Epilepsy with focal impaired consciousness seizures (with observable onset, automatisms)	Oral automatisms occurring during sleep, including smacking and unformed vocalisations, dystonic hand posture on the right side, and impaired consciousness without verbal or nonverbal response.

ECG: electrocardiogram, EEG: electroencephalogram.

## Discussion

Using QSM at 7T, we identified significantly higher susceptibility values across the entire brain in participants with epilepsy compared to healthy controls. The anatomical distribution of brain regions with higher susceptibility colocalised with the presumed seizure onset zone. We found significant correlations between susceptibility and the severity of memory impairment as well as depressive symptoms. Additionally, we observed higher susceptibility postictally as compared to interictally in eight participants with focal epilepsy and the extent of postictal susceptibility changes was linked to the seizure activity before MRI.

At ultra-high magnetic fields, the enhanced susceptibility contrast enables differentiation between tissues based on their iron content,^2^ facilitating the detection of paramagnetic species such as non-haem, extravascular iron. In conditions involving BBB dysfunction, iron may extravasate from the vasculature and accumulate in the parenchyma as free iron, haemosiderin, or ferritin within activated glial cells.^3^ Focal increases in magnetic susceptibility can therefore act as a surrogate marker of prior or ongoing BBB dysfunction.^60^ This underscores the broader applicability of QSM beyond epilepsy, emphasising its potential as a powerful tool for investigating microvascular pathology and iron dysregulation across a wide spectrum of neurological disorders.

Nevertheless, elevated tissue susceptibility should not be assumed to originate solely from extravasated iron. Other factors including inflammation-related metabolic changes, focal epileptogenic lesions, demyelination, calcification, and contributions from diamagnetic tissue constituents can also influence susceptibility measurements.^61^ Although such effects are generally small in cortical and deep grey matter regions,^62^ they remain important considerations for careful interpretation of QSM findings.

The explanation for higher interictal susceptibility in individuals with focal epilepsy relative to healthy controls, is most likely two-fold: Iron accumulation resulting from recurrent epileptic seizures and a generally imbalanced iron homoeostasis in epilepsy.^3^ First, as previously noted, the duration required for iron extravasation following a single seizure to return to baseline—if such normalisation occurs at all—remains uncertain. Considering that repetitive seizures in our study resulted in greater postictal iron accumulation, it is likely that interictal iron levels at least in parts reflect the cumulative impact of previous seizures. Second, iron homoeostasis in the brain is primarily regulated by the BBB. Recent evidence suggests that BBB dysfunction in epilepsy occurs not only during the ictal state but also interictally.^14^ Consequently, individuals with epilepsy likely experience greater iron extravasation than healthy controls in general. Additionally, iron clearance is thought to be mediated by the glymphatic system,^63^ which has also been shown to be compromised in epilepsy.^64^^,^^65^

Extravascular iron accumulation has clinical relevance beyond epileptic seizures. We observed that increased susceptibility was associated with functional impairments in related brain regions. Verbal memory, a marker of left-temporal function, for example, was negatively correlated with susceptibility in the left-temporal cortex and left hippocampus. Episodic memory, which specifically reflects hippocampal function, showed a negative correlation with susceptibility in both hippocampi. Additionally, higher levels of depressive symptoms were linked to increased susceptibility in frontal and default mode network areas, corresponding to regions implicated in major depression.^66^^,^^67^ Iron accumulation in brain tissue can contribute to functional impairment through several neurotoxic mechanisms: Excess iron catalyses the formation of reactive oxygen species, resulting in oxidative stress, lipid peroxidation, and damage to proteins and DNA.^10^^,^^68^ This oxidative environment promotes neuronal and glial injury, disrupts synaptic integrity, and impairs neuroplasticity—processes that are essential for memory, learning, and cognitive function.^69^^,^^70^ The hippocampus and neocortex, which are metabolically active and iron-sensitive regions, are particularly vulnerable to such damage, potentially leading to domain-specific deficits in functions like episodic and verbal memory.^71^ Moreover, iron overload has been shown to activate microglia and promote neuroinflammation, further contributing to neural dysfunction.^72^ Dysregulated iron may, lastly, affect monoaminergic neurotransmitter systems, including dopamine and serotonin, thereby implicating it in the pathophysiology of mood disorders such as depression.^73^^,^^74^

The postictal susceptibility changes we observed are most likely due to ictal BBB dysfunction, allowing iron to permeate into the brain's extracellular matrix.^12^^,^^75^ The extent of ictal iron extravasation, reflected by postictal susceptibility changes, was associated to the seizure activity preceding postictal MRI. Focal to bilateral tonic-clonic seizures led to significantly greater iron extravasation than focal seizures. Additionally, seizure clusters were associated with more iron extravasation than isolated seizures. However, additional factors not captured in our case series, such as the overall condition of the BBB and the individual iron clearance, may affect the extent and duration of postictally measurable ictal iron extravasation. For instance, a pre-damaged BBB from a high seizure burden could result in more extensive and prolonged iron extravasation compared to a first seizure. Future work, focussing on associations between susceptibility alterations and more established, dedicated markers of BBB damage, such as contrast-enhanced imaging, is warranted to further clarify these mechanisms.^12^^,^^14^

Our results suggest two promising avenues for utilising QSM as an imaging biomarker in epilepsy. First, with further development in modelling the relationship between seizure burden and susceptibility values, QSM might become a long-term marker for monitoring seizure burden. Second, given that interictal QSM shows anatomical alignment with the presumed seizure onset zone, it could, if validated further on a single case basis, be employed as a non-invasive imaging modality for localising the seizure onset zone.

This study has two major limitations, one relating to the study cohort and the other to the methodological approach. The first limitation is the heterogeneity within the epilepsy cohort. The presence of pathologies such as focal cortical dysplasias and low-grade epilepsy-associated tumours may influence quantitative susceptibility mapping, for example through diamagnetic calcifications reported in focal cortical dysplasias.^76^ Examples of these lesion types and their appearance on clinical 3T T1-weighted/Fluid-attenuated inversion recovery images and corresponding QSM maps are shown in Supplementary Figure S1. Overlap of presumed seizure onset zones at the group level could further lead to superposition effects, potentially obscuring subtle susceptibility changes in individual cases. Statistical power was limited in the frontal seizure onset subgroup (estimated at ∼45%), which may reduce sensitivity to detect effects in this smaller cohort. In addition, potential confounding factors, such as seizure frequency, duration of illness, and medications exposure, could also influence susceptibility measures. However, seizure frequency is often reported unreliably and specific effects of anti-seizure medications on susceptibility measures remain unknown, rendering formal analysis unfeasible. Collectively, the cohort heterogeneity, overlapping seizure onset zones, limited subgroup power, and unquantified confounders may mask subtle susceptibility changes at the individual level. Finally, sociodemographic factors that may influence biopsychological processes, such as chronic stress associated with socioeconomic status, and which were not captured by our study design, could affect susceptibility values. A second, intrinsically technical limitation of our methodology lies in the fact that, while susceptibility values are widely regarded as a reliable proxy for iron deposition, they can also be influenced by other tissue properties.^77^ Specifically, besides iron, higher susceptibility values can result from a reduction in diamagnetic substances, such as myelin, or an increase in other paramagnetic substances.^77^ Furthermore, our study employed a single, widely used QSM reconstruction method (MEDI). Although we provide a supplementary comparison with an independent method (TGV; Fig. 2) demonstrating consistent results, a broader evaluation across multiple reconstruction techniques could further support and strengthen the present findings in future work.

Interictal iron accumulation shows anatomical colocalisation with the presumed epileptogenic zone and correlates with cognitive deficits and depressive symptoms. Ictal iron extravasation is related to individual seizures. With further validation, QSM could be employed as a non-invasive modality to delineate the epileptogenic zone and potentially serve as a valuable imaging marker for assessing disease severity and quantifying seizure burden.

## Contributors

Nina R. Held (conceptualisation, Data curation, Formal analysis, Investigation, Methodology, Project administration, visualisation, Writing—original draft), Tobias Bauer (conceptualisation, Data curation, Investigation, Funding acquisition, Project administration, Validation, visualisation, Writing—original draft), Rüdiger Stirnberg (methodology, Resources, Software, Writing—original draft), Theda von der Recke (data curation), Nils Lehnen (data curation), Tobias Baumgartner (data curation, Resources, writing–review&editing), Juri-Alexander Witt (data curation, Resources, writing–review&editing) Attila Rácz (data curation), Jan Pukropski (data curation), Lennart Walger (software), Randi D.von Wrede (data curation), Mostafa Badr (data curation), David Wolf (data curation), Lennart Kersting (data curation), Annalena Lange (data curation), Justus Bisten (data curation), Markus Schmidt (data curation), Eberhard Pracht (methodology, Resources), Daniel Löwen (methodology, Resources), Jennifer Faber (resources), Christoph Helmstaedter (resources), Martin Reuter (software), Alon Friedman (supervision), Alexander Radbruch (supervision, Resources, Funding acquisition, writing–review&editing), Rainer Surges (conceptualisation, Supervision, Resources, Funding acquisition, writing–review&editing), Tony Stöcker (conceptualisation, Methodology, Supervision, Resources, Funding acquisition, writing–review&editing), Theodor Rüber (conceptualisation, Data curation, Funding acquisition, Investigation, Project administration, Resources, Supervision, visualisation, Writing—original draft).

All authors read and approved the final version of the manuscript. Nina R. Held, Tobias Bauer and Theodor Rüber have accessed and verified the underlying data.

## Data sharing statement

All data supporting the study can be obtained from the corresponding author upon reasonable request. All code to run the analyses in this can be obtained from the corresponding author upon reasonable request.

## Declaration of interests

J.-A.W. has received payment or honoraria as a speaker from Eisai, UCB Pharma and Jazz Pharmaceuticals. J.F. has received honoraria from Vico therapeutics and Biogen, travel support from ICAR, expenses allowance for lecture for independent neurologists, serves on advisory boards for Vico therapeutics, Biogen and is member of the MDS ataxia study group, the ataxia global initiative steering committee and MR working group. C.H. receives licence fees from UCB Pharma and Eisai, and consulting fees from Angelini and Jazz Pharmaceuticals. R.Surges received grants from the German Federal Ministry of Health, consulting fees from Angelini, LivaNova, Precisis, GmBH, Rapport Therapeutics, UNEEG (payments for advisory boards), as well as received payments for lectures from Angelini, Desitin, Eisai, Jazz Pharmaceuticals, LivaNova, LivAssured, Precisis, Tabuk Pharmaceuticals, UCB Pharma, UnEEG, received travel support from Angelini, serves as chair of the ILAE SUDEP task force and is Associate Editor for Epilepsia Open. N.R.H. received travel bursary from ILAE 2024. A.Racz received Honoria for lectures from University Hospital Essen 2022 and UCB Pharma at the University Hospital Bonn 2023. A.Racz also received travel support from the Elisabeth und Helmut Uhl Stiftung, April 2023, Bolzano, Italy: Launch of the Global Epileptology Research Network. A.Racz received support to attend Stereo-EEG courses (University Hospital Bonn), as well as travel support to attend DGfE congresses 2024, ILAE congresses 2024 and 2025. A.Racz, serves as a Review Editor for the journal Frontiers in Neurology and has received publication fees for previous manuscripts by the Open Access Publication Fund of the University of Bonn. D.W. received support to travel to attend meetings and conferences from Jazz Pharmaceuticals Germany.
