# Supplementary material for: Quantitative susceptibility mapping at 7T as a biomarker of post- and interictal extravascular iron in patients with focal epilepsy
Source: eBioMedicine. 2025 Nov 27;122:106040. doi: 10.1016/j.ebiom.2025.106040 (PMC12703981; doi:10.1016/j.ebiom.2025.106040)
Supplement: Supplementary Figure and Table [file mmc1.pdf]

Supplementary Material for

Quantitative Susceptibility Mapping at 7T as a biomarker of post- and interictal extravascular iron in patients with focal epilepsy

Nina R. Held, Tobias Bauer *et al.*

Corresponding Author Email: theodor.rueber@ukbonn.de

This PDF file includes:

Table S1: pages 2-7
Figure S1: page 8

Table S1. Demographic and clinical characteristics of individuals with focal epilepsy and healthy controls. Age and Age at Onset are reported in five-year ranges. Memory Scores are reported in ten-point ranges. Sex: M = male, F = female. AO = age at onset. VM = verbal memory. FM = figural memory. EM = episodic memory. BDI = Beck's Depression Inventory. SOZ = Seizure Onset Zone. T = temporal. F = frontal. P = parietal. O = occipital. L = Left. R = Right. ASM = total number of antiseizure medications received. SEM = Semiology. iiEEG = interictal scalp-electroencephalography. iEEG = ictal scalp-electroencephalography. NPT = neuropsychological hypothesis. sEEG = stereo-electroencephalography. MRI = Magnetic Resonance Imaging. HS = Hippocampal Sclerosis. VM = Vascular Malformation. AGL = Acquired Gliotic Lesion. LEAT = Low-grade epilepsy-associated Tumour. MCD = Malformation of Cortical Development. MAP = Morphometric Analysis Program. PET = ^{18}F-fluorodeoxyglucose positron emission tomography, SPECT = ictal single-photon emission computed tomography.

ID	Age	Sex	AO	VM	FM	EM	BDI	SOZ	ASM	SEM	iiEEG	iEEG	NPT	sEEG	MRI	MAP	PET	SPECT
1	50-54	M	20-24	70-80	90-100	70-80	7	T	7	Temporal	L Temporal	No ictal pattern	Temporal		Non-Lesional			
2	45-49	M	5-9					T,F	12	L Frontal	L Temporal	L			Non-Lesional			
3	45-49	F	0-4	>100	80-90	90-100		T,F	9	Frontal	L Frontal	L Fronto-temporal	Frontal, R Temporal		L Frontal MCD			
4	30-34	F	5-9	>100	>100	>100	28	T	5	Temporal	L Temporal	L Temporal	Frontal		Non-Lesional		Parietal, Frontal	
5	20-24	F	5-9	>100	90-100	90-100	4	F	6	Unspecific	No abnormal findings	R Frontal	Frontal		L Temporal AGL			
6	55-59	M	5-9	90-100	>100	90-100	5	F,P	12	Frontal	Temporal	L Temporo-parietal	Within normal limits		Non-Lesional		No abnormal findings	Frontal
7	55-59	M	10-14					T,F	9	Fronto-temporal	R Temporal	R Temporal			R HS		R Temporal	
8	20-24	F	0-4	90-100	70-80		7	T,F	4	Unspecific	L Frontal	L Frontal	Frontal, R Temporal		Non-Lesional		No abnormal findings	
9	25-29	F	15-19	>100	>100	>100	5	T,F	8	R Fronto-temporal	Fronto-temporal	R Fronto-temporal	Frontal		Non-Lesional	No abnormal findings	R Frontal	Temporal
10	30-34	M	0-4	60-70		70-80	0	T	11	Temporal	R Temporal	R Temporal	R Temporal		R Temporal AGL		R Temporal	
11	30-34	F	0-4					T	4	Temporal	No abnormal findings	No ictal pattern	L Temporal		L HS			
12	30-34	M	20-24	>100	>100	>100	6	T	5	L Temporal	L Temporal	L Temporal	Within normal Limits	L Temporo-mesial	L HS			
13	35-39	F	0-4	>100	>100	>100	24	T	14	L	L Temporal	Temporal	Frontal	Temporo-mesial	L HS	L Frontal	L Frontal	
14	30-34	F	14-19	90-100	>100	90-100	15	T	3	R Frontal	R Temporal	R Temporal	Within normal limits		R Temporal LEAT		No abnormal findings	
15	35-39	M	14-19	>100	>100		10	T	5	Temporal	R Fronto-temporal	R Temporal	Within normal limits		R Temporal MCD		R Temporal	
16	30-34	F	20-24	70-80	80-90	70-80	29	T	9	Temporal	R Temporal	R Temporal	R Temporal		Limbic Encephalitis	R Temporo-parietal	R Temporal	

Continues on next page

ID	Age	Sex	AO	VM	FM	EM	BDI	SOZ	ASM	SEM	iiEEG	iEEG	NPT	sEEG	MRI	MAP	PET	SPECT
17	25-29	M	5-9	90-100	90-100	90-100	8	F	4	Frontal	L Frontal	L Frontal	Frontal		L Frontal MCD		L Frontal	
18	40-44	M	0-4	>100	80-90	80-90	5	T	7	R Fronto-temporal	L Temporal	L Temporal	Temporal		Hypo-thalamic Hamartoma		No abnormal findings	
19	20-24	M	15-19	>100	>100	>100	6	F	6	Frontal	R Frontal	R Frontal	Within normal limits	R Frontal	R Temporal AGL	R Temporal	R Frontal	Frontal
20	25-29	M	5-9		80-90	70-80	16	T	8	Temporal	L Temporal	R Temporal	Unspecific	L Temporal	Limbic Encephalitis	No abnormal findings	L Temporal	Temporal
21	35-39	F	14-19					F	4	Fronto-temporal	R Frontal	R Frontal			Non-Lesional			
22	25-29	M	14-19	60-70	50-60	50-60	5	T	10	Temporal	L Temporal	R Temporal	Unspecific		Multiple MCD		R Temporal	
23	55-59	F	40-44	>100	>100	>100	27	T	2	Frontal	Temporal	R Temporal	Within normal limits		Limbic Encephalitis		R Temporal	
24	35-39	M	10-14	80-90	>100	90-100	7	T	5	Temporal	L Frontal	L Frontal	L Temporal		L Temporal MCD		Frontal	L Temporal
25	30-34	M	15-19					T,F	5	Frontal	R Fronto-temporal	R Fronto-temporal			L HS		Temporo-parietal	
26	20-24	M	10-14	>100	90-100	90-100	9	T,F	5	Temporal, Frontal	Fronto-temporal	L Frontal	Frontal		Non-Lesional	R Frontal	No abnormal findings	Fronto-temporal
27	20-24	M	15-19	80-90	90-100	80-90	31	T	6	Temporal	L Fronto-temporal	Temporal	L Temporal		Non-Lesional		Fronto-parieto-temporal	
28	30-34	F	15-19	50-60	50-60	60-70	36	T	3	Temporal	R Temporal	R Temporal	Temporal	R Temporal	Non-Lesional		Fronto-temporal	R Temporal
29	<20	M	10-14	90-100	90-100	90-100	4	F	4	Frontal	L Frontal	L Frontal	L Frontal		Non-Lesional		Unspecific	
30	20-24	M	0-4	90-100	>100	>100	10	T,F	4	Fronto-temporal	L Frontal	L	L Fronto-temporal		Non-Lesional	No abnormal findings	R Fronto-temporal	L
31	20-24	M	15-19	60-70	>100	70-80	1	T	3	L Temporal	L Temporal	L Temporal	L Temporal		Limbic Encephalitis			
32	20-24	F	15-19	90-100	90-100	90-100	10	T	4	Temporal	R Frontal	No ictal pattern	Frontal		Non-Lesional			
33	70-74	F	65-69	90-100	80-90	80-90	1	T	1	Temporal	Temporal	Temporal	Fronto-temporal		R Temporal MCD			
34	25-29	M	5-9					F	8	Frontal	No abnormal findings	No ictal pattern			Non-Lesional		R Frontal	R Frontal
35	20-24	F	15-19	70-80	60-70	60-70	41	T,P	6	R Temporal	R Temporal	R Temporo-parietal	Temporal	R Temporo-parietal	R Parietal MCD		R Temporal	
36	35-39	M	30-34	>100	>100	>100	4	T	3	L Temporal	Temporal	L Temporal	Within normal limits		Frontal AGL		L Temporal	
37	30-34	M	20-24	>100	>100	>100	8	T	4	R Fronto-temporal	R Fronto-temporal	R Fronto-temporal	Frontal	R Temporal	R Temporal MCD		R Temporal	

Continues on next page

ID	Age	Sex	AO	VM	FM	EM	BDI	SOZ	ASM	SEM	iiEEG	iEEG	NPT	sEEG	MRI	MAP	PET	SPECT
38	30-34	M	30-34	80-90	70-80	70-80	4	T	2	Temporal	No abnormal findings	L Temporal	Temporal		L Temporal LEAT, Limbic Encephalitis		L Temporal	
39	25-29	M	20-24	90-100	70-80	80-90		T,F,P	5	Unspecific	R Temporal	Bilateral	Unspecific		Non-Lesional		Frontal, R Temporo-parietal	
40	30-34	F	25-29					T,O	3	Temporal	No abnormal findings	Generalised ictal pattern			L Occipital VM		L Occipital	
41	25-29	F	10-14	90-100	70-80	70-80	5	T	10	Temporal	R Temporal	R Temporal	Temporal	R Temporal	L HS		Temporal	Temporal
42	20-24	M	15-19	90-100	70-80	80-90	20	F	5	L Frontal	L Frontal	L Frontal	Unspecific	L Frontal	L Frontal MCD, Cerebellar Lesion	L Frontal	L Fronto-temporal	
43	<20	F	15-19	>100	60-70	80-90	16	T	2	L Temporal	L Temporal	L Temporal	R Temporal		L Temporal VM		L Temporal	
44	30-34	F	25-29					T	6	R Temporal	R Temporal	R Temporal	R Temporal		Non-Lesional		R Temporal	
45	<20	M	10-14					F	2	L	Frontal	Frontal			Non-Lesional		No abnormal findings	
46	25-29	M	10-14	80-90	80-90	70-80		T,F	8	L	L Temporal	Bilateral	Fronto-temporal		Non-Lesional		L Temporal	
47	<20	M	10-14					F	2	Frontal	No abnormal findings	No ictal pattern			Non-Lesional			
48	30-34	F	0-4		60-70	70-80		T,P	3	Frontal	No abnormal findings	R Parietal	Frontal, R Temporal		R HS		R Occipital	
49	20-24	M	10-14	80-90	90-100	80-90		T,F	5	Fronto-temporal	Frontal	Frontal	Unspecific	Hypo-thalamic, Fronto-temporal	Hypo-thalamic Hamartoma		Frontal	
50	40-44	M	10-14	80-90	80-90	80-90	9	T	3	L Temporal	L Fronto-temporal	L Temporal	Fronto-temporal		L Temporal MCD		Temporal	
51	45-49	M	25-29	80-90	80-90	70-80	17	T,F	11	L Temporal	No abnormal findings	Fronto-temporal	Fronto-temporal		Left Parietal AGL		Frontal	
52	25-29	F	10-14	70-80	80-90	80-90	45	T	10	Temporal	L Temporal	L Temporal	L Temporal		Left HS		L Temporal	
53	40-44	M	20-24	90-100	80-90	90-100	11	F	4	L Frontal	No abnormal findings	L Fronto-temporal	R Fronto-temporal		Non-Lesional			
54	20-24	F	0-4		70-80	70-80	5	T	10	R	R Temporal	R Temporal	R Temporal	R Temporal	R Temporal MCD		R Temporo-parietal	
55	40-44	F	40-44	80-90	70-80	70-80	17	T	3	Temporal	No abnormal findings	L Temporal	Within normal limits		L Temporal LEAT		No abnormal findings	
56	35-39	M	10-14	80-90	60-70	70-80		F	6	Fronto-temporal	No abnormal findings	L Frontal	Unspecific		Non-Lesional			

Continues on next page

ID	Age	Sex	AO	VM	FM	EM	BDI	SOZ	ASM	SEM	iiEEG	iEEG	NPT	sEEG	MRI	MAP	PET	SPECT
57	30-34	F	15-19	90-100	70-80	80-90	13	F	3	Frontal	L Temporal	No ictal pattern	Unspecific	R Frontal	R Frontal MCD		No abnormal findings	No abnormal findings
58	40-44	M	20-24	90-100	80-90	90-100	15	T	7	R Temporal	R Temporal	R Temporal	R Temporal		R Temporal VM		R Temporal	
59	50-54	M	45-49	90-100	90-100		6	T	2	Temporal	L Temporal	L Temporal	L Temporal		Non-Lesional		L Temporal	
60	40-44	M	5-9	>100	90-100	90-100	16	T,F	9	Frontal	L Temporal	L Fronto-temporal	Within normal limits		Non-Lesional		No abnormal findings	
61	35-39	M	10-14					F	11	Frontal	Frontal	Frontal			Non-Lesional			Frontal
62	<20	M	10-14	60-70	70-80	60-70		T	4	L Temporal	L Temporal	L Temporal	Temporal		L HS		L Temporal	
63	40-44	F	15-19					T	2	R Temporal	R Temporal	R Temporal			Non-Lesional		Unspecific	
64	30-34	F	0-4	70-80	80-90	70-80	26	T	7	L Temporal	L Temporal	L Temporal	L Temporal		L Temporal LEAT		L Temporal	
65	45-49	M	30-34	60-70	70-80	70-80	0	T	4	Temporal	L Temporal	L Temporal	Temporal		L Temporal MCD, L-Hemispheric Atrophy		L	
66	30-34	M	5-9	90-100	80-90	90-100	9	F	5	R Frontal	R Frontal	R Frontal	Frontal	R Frontal	R Frontal MCD		No abnormal finding	
67	25-29	F	15-19	>100	80-90		8	T,F	6	Frontal	R Frontal	R Frontal	R Fronto-temporal	R Frontal	L AGL		R Frontal	
68	45-49	F	0-4	80-90	70-80		32	T	5	Unspecific	L Temporal	No ictal pattern	Unspecific		L HS		L Temporal	
69	20-24	F	10-14	>100	>100		20	T	6	L Temporal	Unspecific	L Temporal	L Temporal	L Temporal	L Temporal AGL		L Temporal	
70	<20	F	0-4	80-90	80-90	80-90	12	T,F	9	L	L Fronto-temporo-occipital	L Fronto-temporal	Frontal		Non-Lesional		L Temporal	
71	25-29	F	20-24	>100	>100	>100	2	T	4	Temporal	No abnormal findings	L Temporal	L Fronto-temporal		Limbic Encephalitis		L Temporal	
72	20-24	F	10-14	80-90	90-100	80-90	6	T	4	L	L Temporal	L Temporal	L Temporal		L Temporal AGL		Temporal	
73	45-49	F	10-14	>100	>100	>100	3	T	9	R Temporal	R Temporal	R Temporal	Within normal limits		R Temporal MCD		R Temporal	
74	35-39	F	10-14	70-80	80-90		8	F	7	Frontal	R Frontal	R Frontal	Fronto-temporal	R Frontal	L Frontal MCD		Frontal	L Frontal
75	25-29	F	15-19	70-80	>100	80-90	8	T	11	L Temporal	Temporal	L	L Temporal		L Temporal LEAT, Multiple MCD		L Temporal	
76	25-29	M	0-4	>100	90-100	90-100	11	T	18	R	Temporal	R Fronto-temporal	Frontal		Non-Lesional		R Temporal	
77	40-44	M	25-29	60-70	70-80			F	4	R Frontal	R Frontal	R Frontal	Unspecific		VM		R Frontal	
78	45-49	M	35-39	70-80	70-80	70-80	24	T	10	Fronto-temporal	L Temporal	No ictal pattern	R Temporal	L Temporal	Non-Lesional		R Temporal	R Temporal

Continues on next page

ID	Age	Sex	AO	VM	FM	EM	BDI	SOZ	ASM	SEM	iiEEG	iEEG	NPT	sEEG	MRI	MAP	PET	SPECT
79	30-34	M	20-24	>100	>100	>100	18	F	2	Frontal	R Frontal, L Temporal	R Frontal	L Temporal		Non-Lesional		R Fronto-temporal	
80	35-39	F	5-9	90-100	90-100	80-90	4	T	13	Fronto-temporal	L Temporal	No ictal pattern	L Fronto-temporal		Non-Lesional		R Temporal	
C1	20-24	M																
C2	35-39	F																
C3	40-44	M																
C4	20-24	M																
C5	30-34	M																
C6	20-24	M																
C7	25-29	F																
C8	25-29	F																
C9	20-24	F																
C10	25-29	F																
C11	25-29	M																
C12	25-29	F																
C13	25-29	M																
C14	20-24	F																
C15	25-29	M																
C16	25-29	M																
C17	30-34	M																
C18	30-34	F																
C19	45-49	M																
C20	70-74	M																
C21	35-39	F																
C22	40-44	F																
C23	25-29	M																
C24	25-29	M																
C25	20-24	M																
C26	20-24	F																
C27	25-29	M																
C28	25-29	F																
C29	20-24	M																
C30	20-24	M																
C31	30-34	F																
C32	20-24	M																
C33	20-24	F																
C34	20-24	F																
C35	25-29	F																
C36	25-29	F																
C37	25-29	F																
C38	25-29	F																
C39	30-34	M																
C40	25-29	F																
C41	25-29	M																
C42	20-24	F																
C43	25-29	F																
C44	<20	F																
C45	25-29	M																
C46	25-29	F																

Continues on next page

ID	Age	Sex	AO	VM	FM	EM	BDI	SOZ	ASM	SEM	iiEEG	iEEG	NPT	sEEG	MRI	MAP	PET	SPECT
C47	25-29	M																
C48	25-29	M																
C49	25-29	M																
C50	30-34	M																

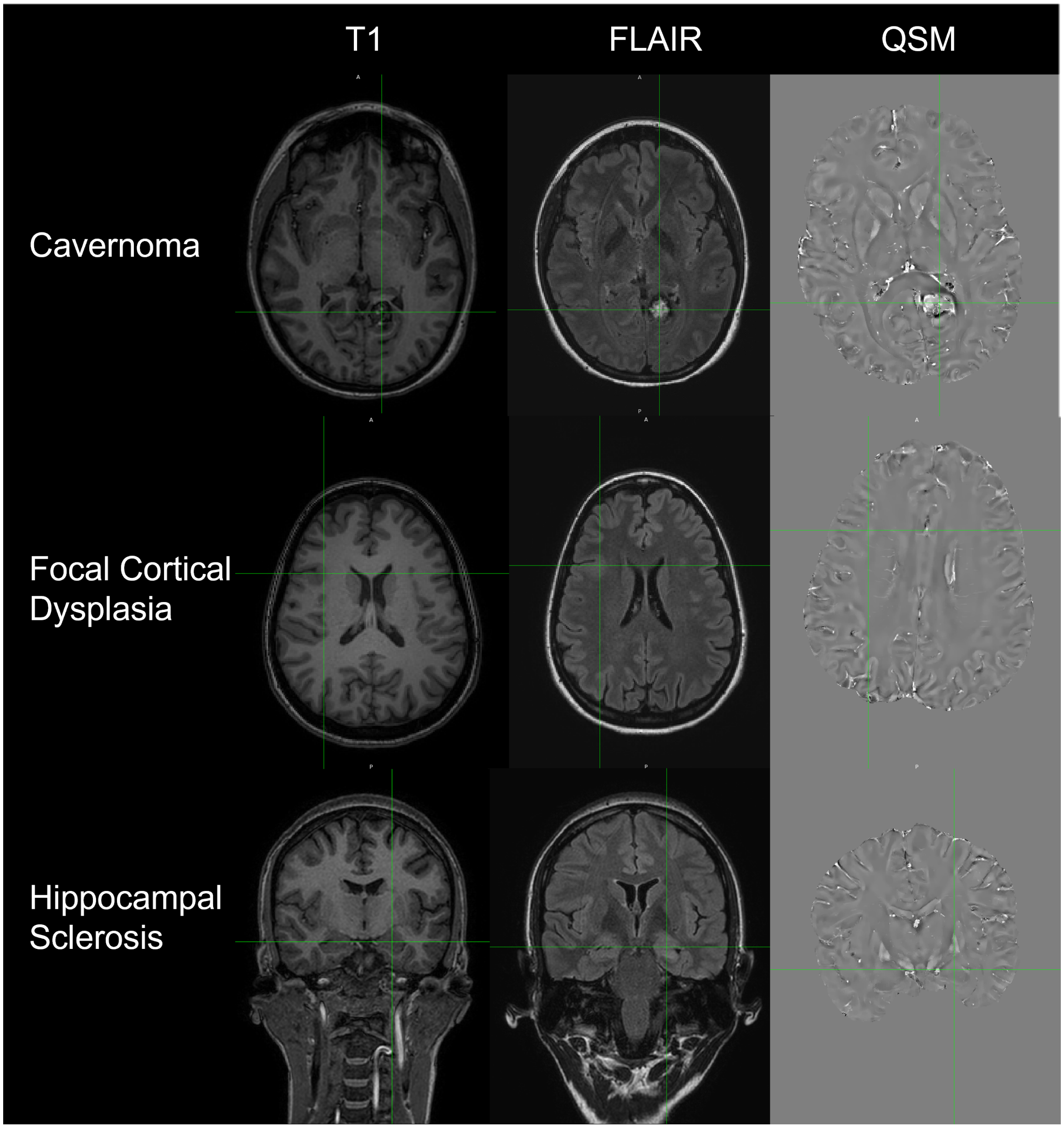

Figure S1. Lesion examples in subjects with focal epilepsy. For each case, clinical 3 Tesla T1-weighted and Fluid-attenuated inversion recovery images are shown alongside quantitative susceptibility maps (values: –0·2 to 0·2 ppm). From top to bottom: cavernoma, focal cortical dysplasia and hippocampal sclerosis.
